# Use of Virtual Reality for Pediatric Cardiac Critical Care Simulation

**DOI:** 10.7759/cureus.15856

**Published:** 2021-06-23

**Authors:** Bradford H Ralston, Renee C Willett, Srihari Namperumal, Nina M Brown, Heather Walsh, Ricardo A Muñoz, Sylvia del Castillo, Todd P Chang, Gregory K Yurasek

**Affiliations:** 1 Division of Cardiology, Children's National Hospital, Washington, DC, USA; 2 Cardiac Critical Care, Children's National Hospital, Washington, DC, USA; 3 Division of Cardiac Critical Care, George Washington University, Washington, DC, USA; 4 Emergency Medicine, The Permanente Medical Group, San Leandro, USA; 5 Simulation Center, Children's National Hospital, Washington, DC, USA; 6 Cardiac Critical Care, Children's Hospital Los Angeles, Los Angeles, USA; 7 Critical Care Anesthesiology, Keck School of Medicine at University of Southern California, Los Angeles, USA; 8 Emergency Medicine, Children's Hospital Los Angeles, Los Angeles, USA; 9 Division of Emergency Medicine, Keck School of Medicine at University of Southern California, Los Angeles, USA

**Keywords:** pediatric cardiac intensive care, virtual reality simulation, pediatric cardiology, junctional ectopic tachycardia, covid-19 infection control, intubation simulation, pediatric cardiovascular surgery

## Abstract

Simulation is a key component of training in the pediatric cardiac intensive care unit (CICU), a complex environment that lends itself to virtual reality (VR)-based simulations. However, VR has not been previously described for this purpose. Two simulations were developed to test the use of VR in simulating pediatric CICU clinical scenarios, one simulating junctional ectopic tachycardia and low cardiac output syndrome, and the other simulating acute respiratory failure in a patient with suspected coronavirus disease 2019. Six attending pediatric cardiac critical care physicians were recruited to participate in the simulations as a pilot test of VR’s feasibility for educational and practice improvement efforts in this highly specialized clinical environment. All participants successfully navigated the VR environment and met the critical endpoints of the two clinical scenarios. Qualitative feedback was overall positive with some specific critiques regarding limited realism in some mechanical aspects of the simulation. This is the first described use of VR in pediatric cardiac critical care simulation.

## Introduction

The pediatric cardiac intensive care unit (CICU) is a high-acuity environment. Many patients have limited physiologic reserve, and any acute changes in their physiology need to be managed promptly to prevent cardiac arrest [1,2]. With an ever-increasing culture of safety and increased oversight, trainees working in this environment may not receive sufficient experiential training in personally managing events of this nature, and thus may not be adequately prepared to act autonomously when such events occur [3-6]. Simulation-based training allows trainees to learn in a safe learning environment, and plays a critical role in pediatric CICU education and training [3,7-9]. While conventional mannequin-based simulation can be a useful tool, upkeep and maintenance of high-fidelity simulators is expensive, and they can be cumbersome to set up and operate [10,11]. Virtual reality (VR) is an alternative simulation methodology being used increasingly in medical training and education [12-16]. VR has the potential advantage of increased “immersion,” the sense of being physically in the virtual space or performing a task, and has been shown to manifest physiologic stress responses akin to real life among physicians participating in resuscitation simulations [13,17]. It is well suited for use in the CICU where mannequin setup involves a complex array of vascular access lines, mechanical ventilators, and physiologic monitors, as well as mannequins of varying sizes in pediatrics, all requiring a great deal of time to set up in a realistic manner. VR-based simulation circumvents these issues but has not been previously described as a tool for simulation in the pediatric CICU. The purpose of this manuscript is to describe our initial experience in developing two pilot VR scenarios and to demonstrate its feasibility in highly specialized clinical environments.

## Materials and methods

Pediatric cardiology and critical care fellows as well as advanced practice providers and bedside nursing staff participate in periodic mannequin-based in situ simulations in the CICU at our institution for the purposes of education and practice improvement. The frequency of such simulations is limited by logistics of mannequin setup, bed space availability, and clinician availability, and generally only a single learner serves in the role of the team leader in each session due to time constraints. We planned a series of VR simulation scenarios for the purposes of training fellows and advanced practice providers in the pediatric CICU that can be easily set up and implemented for several learners consecutively or simultaneously, with opportunities for repetition and iterative practice. An immersive pediatric CICU environment was designed to replicate a realistic setting by case scenario authors who regularly practice in the pediatric CICU (BR, GY, and RW). Two pilot scenarios were designed and programmed as a proof of concept to demonstrate the efficacy of VR to simulate and teach specialized skills required for practice in the CICU. The medical decision-making logic, clinical objectives, and clinical feedback data were developed and mapped in detail by the case scenario authors. All programming and coding were performed by the medical VR simulation development company SimX (San Francisco, CA, USA) with an iterative revision process in cooperation with the case authors over a six-month period. Simulations were run on the SimX platform using Oculus Quest head-mounted displays (Oculus from Facebook, San Jose, CA, USA).

The first scenario required participants to correctly recognize and treat junctional ectopic tachycardia and low cardiac output syndrome (JET/LCOS) in the early postoperative period for a patient after congenital heart surgery. This scenario was selected as a pilot simulation in order to test the ability of participants to interpret the wide array of vital sign changes and other patient parameters that trainees would be expected to encounter. This simulation was also chosen to test the interplay among multiple complex physiological parameters with multiple branching-points in the case progression algorithm. The second scenario, in contrast, required participants to follow proper infection control procedures in the process of intubating a patient with suspected coronavirus disease 2019 (COVID-19)-associated respiratory failure (COVID Intubation). This scenario was selected to investigate the utility of VR in rehearsing a stepwise protocol rather than simulate complex medical decision-making. These two scenarios were chosen specifically to encompass a variety of clinical tools and processes in order to identify aspects of the simulations that are particularly effective as well as those that may be problematic in developing additional scenarios. They were intentionally designed to differentially emphasize aspects of physical fidelity (with different equipment and environmental details in the two scenarios), psychological fidelity (complex decision-making in LCOS/JET vs linear cognitive processes in COVID Intubation), and functional fidelity (complex physiological feedback in JET/LCOS vs planning and team communication in COVID Intubation). Predetermined endpoints were established in the scenario designs such that the scenario would conclude when either critical endpoints were successfully met, or the patient critically deteriorated based upon the programmed algorithm. Details of the scenario designs are outlined in Table 1.

**Table 1:** Clinical case scenarios VR, virtual reality; EMR, electronic medical records; COVID-19, coronavirus disease 2019; PPE, personal protective equipment; PAPR, power air-purifying respirator; JET, junctional ectopic tachycardia; NIRS, near-infrared spectroscopy; HEPA, high-efficiency particulate air; EMS, emergency medical services.

	Junctional Ectopic Tachycardia/Low Cardiac Output Syndrome	Acute Respiratory Failure Due to Suspected COVID-19 Infection
Clinical case overview	A three-month old on postoperative day 1 status post repair of a complete atrioventricular canal defect with new-onset tachycardia	A six-month old with a history of a repaired atrioventricular canal defect presents with acute respiratory failure in the setting of recent exposure to COVID-19
Prebriefing components	-Orientation to the VR headset and hand controls	-Orientation to the VR environment
	-Orientation to the virtual clinical environment	-Disclosure of the suspected diagnosis and learning goals
	-Orientation to physiological monitors, data that can be obtained by examination, and data that can be obtained in the virtual EMR	-Review of institutional guidelines and instructions regarding safe COVID-19 airway management
	-Overview of mechanisms for interacting (patient examination, interactions with tools including pacemaker, ventilator, medication pumps, and so on)	-Clinical history (provided by virtual characters including transport team paramedic and bedside nurse)
	-Limited clinical history as per the overview without specifics regarding the acute diagnosis or learning goals	-Verbal instructions from the moderator regarding available tools specific to the case (airway supplies and so on)
Case-specific tools, features, and media	-Postoperative intubated infant with post-sternotomy dressing, pacing wires, and vascular access	-Two-room setup with some actions occurring in the space outside the patient’s room, and the second part of the simulation occurring inside the patient room
	-Detailed physiologic monitors including arterial and central venous pressure tracings	-Wearable PPE including gloves, N95 mask, isolation gown, face shield, and PAPR
	-Monitor tracings to represent different physiologic representations of JET (absent P waves, peaked A waves, dampened arterial line tracing) vs pacing (pacing spikes with the heart rate matching the set paced rate)	-Physiologic monitors that function only when specific monitors are connected to the patient (pulse oximeter, EKG leads)
	-Vital sign trends monitor demonstrating trends in heart rate, blood pressure, oxygen saturation, NIRS, central venous pressure, and relative oxygen delivery deficit	-Airway supplies including bag-valve mask, suction, endotracheal tube, conventional laryngoscopy blade, and a ventilator with adjustable settings
	-Patient temperature control buttons to adjust radiant warmer and cooling blanket	-Video laryngoscope with side display of the patient’s airway with and without an endotracheal tube in place
	-Temporary pacemaker with adjustable knobs to control output parameters	-HEPA filter to be attached to the endotracheal tube
	-Medication infusion pumps with multiple running medications and several others “in line” to be used as needed	-Walkie-talkie to communicate with staff outside the patient’s room
	-Surface and atrial ECGs demonstrating junctional ectopic tachycardia	-Several characters with extensive dialogue to interact with the participant (EMS provider, patient’s mother, two nurses)
	-Virtual bedside nurse and a parent with triggerable dialogue pertinent to the case	
Case progression	Participant should recognize junctional ectopic tachycardia and low cardiac output syndrome; an atrial ECG should be ordered to confirm the diagnosis. Intravascular volume expansion, sedation, and cooling the patient should be implemented, and then the patient should be connected to the temporary pacemaker and overdrive paced once the heart rate has decreased. The scenario then concludes. Increasing inotropes will have a temporizing effect on blood pressure but will worsen tachycardia and prevent optimizing the heart rate for overdrive pacing. Excessive sedation, inaction, or management of the wrong condition results in cardiac arrest and the scenario concludes.	The scenario begins outside the patient’s negative pressure room where the history is provided, and the participant should determine that they will need to intubate the patient using COVID-19-safe procedures. The participant should conduct a time-out before entering the patient’s room to discuss supplies and medications that will be needed and don virtual PPE. After entering the room, the participant should examine the patient and make all preparations to intubate the patient including patient positioning and gathering of supplies. The participant can communicate with virtual nurses outside of the room using a walkie-talkie. The patient should then be sedated, muscle-relaxed, and intubated using the video laryngoscope using appropriate precautions to avoid particle aerosolization.
Checklist items	-Administer volume resuscitation	-Conduct a time-out before entering the patient room; request a ventilator, video laryngoscope, and medications
	-Cool the patient	-Properly don PPE
	-Administer sedation and/or neuromuscular blockade	-Avoid unnecessary bag-mask ventilation
	-Request an atrial ECG	-Use the video laryngoscope to intubate the patient
	-Connect the patient to the pacemaker and correctly overdrive pace	-Connect a HEPA filter to the endotracheal tube before bagging or connecting to the ventilator
Critical steps to end the scenario	Connect the patient to the pacemaker and correctly overdrive pace	Intubate the patient and connect to the ventilator

After obtaining Institutional Review Board approval, six attending pediatric CICU physicians were enrolled. As our objective was to solicit feedback regarding strengths and weaknesses of the simulation experience itself rather than to identify clinical knowledge gaps, experienced attending physicians were selected for this initial study. None of the participants had prior experience with VR-based simulation training.

Each physician participated as a single provider in the simulation, and they were blinded to the clinical scenarios until the beginning of each simulation. The participant was first oriented to the VR environment as well as to the headset and hand controls. After adequate prebriefing and an opportunity to ask questions about navigating the VR environment, the participant was then asked to individually manage the virtual patient in the two clinical scenarios. Clinical information was obtained by the participant within the scenario itself whenever possible rather than by having it provided by the moderator. For example, information was obtained by physically looking at virtual monitors in the environment, looking through a simulated tablet-based electronic medical record in the environment, or by examining the virtual patient to receive tactile, visual, and audible feedback in response to the participant’s exam maneuvers and medical treatments. Additional information could be obtained through dialogue with virtual characters embedded into the simulation environment, each with preprogrammed responses to anticipated questions.

**Figure 1:**
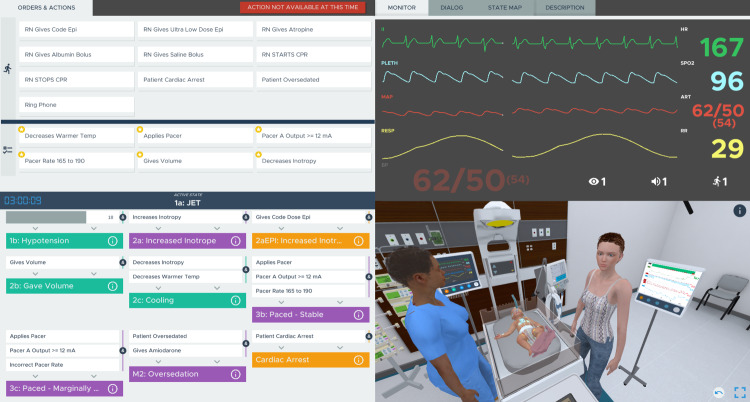
Interactive moderator display during the JET/LCOS scenario The moderator’s screen divided into four quadrants demonstrates actions that can be manually activated or automatically triggered by the participant's actions (top left), real-time vital sign display (top right), a map of the case progression (bottom left), and a mirror-streamed two-dimensional projection of the participant’s view. JET/LCOS, junctional ectopic tachycardia and low cardiac output syndrome.

Case scenario authors served as moderators and observed the participant’s management in real time via a mirror-streamed display. The moderator’s display was augmented with additional technical information regarding the simulation’s logic and stage progression as well as controls to manually trigger responses to the participant’s actions along with dialogue buttons for the embedded virtual characters (Figure 1). Manual controls were used only secondarily, as the cases were designed to progress and respond to the participants’ actions automatically without manual control from the moderator except when engaging in dialogue with simulated characters, or when the participant deviates substantially from the expected management strategy. The observers maintained checklists of the management objectives met by each participant. Scenarios concluded when the predetermined endpoints were met.

A targeted debrief was conducted after each session for each participant in efforts to provide bidirectional feedback. This encompassed both feedback to the participants regarding their performance in diagnosing and managing the clinical scenarios as well as comments from participants regarding their perceptions of the simulation experience. After the second debrief was complete, the participant was asked to complete a six-question survey regarding their experience. Answers were rated on a five-point Likert scale, and additional space was provided for other comments.

## Results

**Figure 2:**
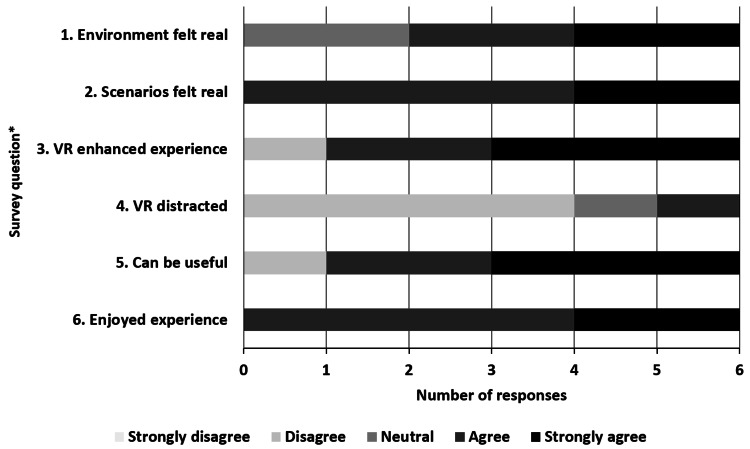
Participant survey responses *Survey questions: 1. The VR environment felt realistic. 2. The clinical scenarios were realistic and simulated my experiences in real-life situations. 3. VR enhanced my simulation experience. 4. The VR medium distracted from medical decision-making. 5. VR-based simulation can be useful for education in pediatric cardiac intensive care. 6. I enjoyed the experience. VR, virtual reality.

Six participants completed both pilot scenarios. All participants completed post-participation surveys, and all six agreed or strongly agreed that they “enjoyed the experience” (Figure 2).

Four out of six reported they “agree” or “strongly agree” that the VR environment felt realistic, while all six participants agreed or strongly agreed that the clinical scenarios were realistic and representative of real-life situations.

Five out of six agreed or strongly agreed that VR enhanced the simulation experience, with one participant disagreeing, adding the comment “at least not yet.” Another commented that the effect of the experience was overall “similar to” their experience with other types of simulation technology. Four out of six felt that the VR medium did not distract from medical decision-making, while one felt neutral, and the final participant reported that while it is currently a distraction, “with more practice it probably won’t [be].” All but one participant agreed or strongly agreed that VR can be useful for education in the pediatric CICU. No participants had to pause or stop a scenario prematurely due to adverse sensations such as nausea or disorientation.

All six participants correctly recognized the diagnosis in the JET/LCOS scenario and completed the critical objective of overdriving pacing the patient in a median of 6 minutes (range 4-8 minutes). Of the five checklist objectives, most participants met all five (range 3-5), with the most frequently missed item being cooling the patient. Both participants who did not meet this objective did, however, administer sedation and paralytic, which has a similar clinical effect and may be considered a reasonable alternative in clinical practice.

In the COVID Intubation scenario, all five objectives were met by all participants except one who did not conduct a time-out before entering the patient room.

## Discussion

VR-based simulation was successfully applied to two different types of cases simulating realistic clinical scenarios in a pediatric CICU. Experienced attending physicians familiar with the simulated medical conditions were able to successfully navigate the VR environment and complete the simulation exercises. While there was some variation in the specifics of the clinical management, as would be expected in actual clinical practice, all participants met the critical endpoint objective in both scenarios, demonstrating feasibility of VR for this purpose. Feedback from the participants was overall positive with some specific critiques and reservations, but nearly all felt that VR enhanced their simulation experience.

One specific success in this pilot activity was the ability of the participants to diagnose and manage JET in the LCOS case as this required attention to a number of patient parameters in a nuanced manner. Participants naturalistically processed familiar data as it was presented to them including the rhythm on the monitor, the vital sign trend data showing an acute increase in the heart rate, the rising central venous pressure with an abnormal pressure tracing, and the declining near-infrared spectroscopy value at the start of the case. They responded fluidly as the physiology changed in response to their actions as the case progressed. With each intervention, participants habitually reassessed the data being presented to plan their next steps, demonstrating the functional fidelity of the simulation. Most participants employed all of the expected management strategies to treat the VR patient, and all met the critical objective of correctly overdrive pacing the patient. This suggests that VR may sufficiently simulate real-life scenarios with complex physiology to facilitate training and assess learners’ comprehension of physiology and capacity to manage patients in the pediatric CICU. Moreover, the time to complete this scenario was relatively brief. With essentially no turnover time, a facilitator can reset the scenario to permit learners to repeat or restart a scenario in order to reinforce particular steps in management or try a different approach to allow for flexible learning.

The COVID Intubation case demonstrated the utility of using VR to simulate linear processes and protocols. This appeared to be effective as a means of reinforcing the specific steps practitioners would need to take in managing a critically ill patient with respiratory failure in the COVID era. The major limitation in this scenario was that participants did not find the exercise realistic in simulating the mechanical processes of equipment management for endotracheal intubation. While practicing the actual skill of endotracheal intubation was not the educational objective of this simulation, the procedure’s limited fidelity did appear to detract from the intended objectives. This may have been partly related to inherent limitations of haptic and kinetic feedback using conventional VR equipment, which would be difficult and costly to effectively overcome. However, the participants’ comments suggest that the larger issue was that the simulated equipment could not be moved around the room and positioned spontaneously to set up for the procedure, a functional limitation that could be addressed with relatively simple software and programming adjustments. This opportunity for improvement is supported by previous literature that has described the successful use of VR to simulate pediatric airway intubation [18].

VR-based simulation has been described extensively for procedural training in several surgical specialties [19-24]. In contrast, our goal was primarily to simulate the less mechanical aspects of managing a pediatric CICU emergency, with emphasis on data synthesis and cognitive processes. Though VR has been described as a tool for simulating various nontechnical skills within the realm of medical training, there is a paucity of data on efficacy with this type of learning [13,25]. Our work contributes to this growing body of literature by demonstrating feasibility in a new setting, but with more complex logic and physiologic interactions than previously described applications. With additional work demonstrating noninferiority (if not superiority) of VR to other types of simulation in the pediatric CICU, the potential advantages of VR over mannequin-based simulation, such as decreased maintenance costs and simplified setup logistics, would render VR a key modality for future training.

## Conclusions

In summary, our initial experience demonstrates that with minimal orientation, clinicians previously unfamiliar with VR can engage in simulated acute clinical scenarios common to the pediatric CICU. To our knowledge, this is the first use of VR for simulation in the pediatric CICU. Diagnostic data and hemodynamic feedback were simulated with ample fidelity for experienced physicians to recognize and manage the two conditions. Moving forward, we plan to implement these and additional simulated clinical scenarios into the educational curriculum for clinical trainees and nurses. Future studies are needed to evaluate VR’s utility as a learning and teaching tool in this environment, and to compare the performance and efficiency of VR with mannequin-based simulation training with the ultimate goal of improved patient safety and care delivery through a more knowledgeable and prepared critical care staff.
